# Sex difference in the incidence of microvascular complications in patients with type 2 diabetes mellitus: a prospective cohort study

**DOI:** 10.1007/s00592-020-01489-6

**Published:** 2020-02-05

**Authors:** Sunny S. Singh, Jeanine E. Roeters-van Lennep, Roosmarijn F. H. Lemmers, Thijs T. W. van Herpt, Aloysius G. Lieverse, Eric J. G. Sijbrands, Mandy van Hoek

**Affiliations:** 1 Department of Internal Medicine, Erasmus MC - University Medical Center Rotterdam, Rotterdam, The Netherlands; 2 Department of Internal Medicine, Maxima Medical Center, Eindhoven, The Netherlands; 3 Department of Internal Medicine, Amphia Hospital, Breda, The Netherlands

**Keywords:** Sex differences, Microvascular complications, Retinopathy, Microalbuminuria, Neuropathy

## Abstract

**Aims:**

Type 2 diabetes mellitus is a major cause of death and disability due to its long-term macro- and microvascular diseases. Although women with type 2 diabetes have more macrovascular diseases, it is unclear whether there are sex differences in the occurrence of microvascular disease. The aim of our study was to investigate sex differences in the incidence of microvascular complications in type 2 diabetes.

**Methods:**

Analyses were performed in the DiaGene study, a prospective cohort study for complications of type 2 diabetes, collected in the city of Eindhoven, the Netherlands (*n* = 1886, mean follow-up time = 6.93 years). Cox proportional hazard models adjusted for risk factors for complications (age, smoking, hypertension, dyslipidemia, HbA1c and duration of type 2 diabetes) were used to analyze the incidence of microvascular complications in men and women.

**Results:**

The incidence of microalbuminuria was significantly higher in men (HR microalbuminuria 1.64 [CI 1.21–2.24], *p* = 0.002). Additionally, men are more likely to develop two or three microvascular complications compared to women (OR 2.42 [CI 1.69–3.45], *p* < 0.001).

**Conclusions:**

This study shows that men with type 2 diabetes are more likely to develop microvascular complications, especially microalbuminuria. Furthermore, men seem to have a higher chance of developing multiple microvascular complications. Our results highlight that men and women may not benefit to a similar extent from current treatment approaches to prevent diabetes-related microvascular diseases.

**Electronic supplementary material:**

The online version of this article (10.1007/s00592-020-01489-6) contains supplementary material, which is available to authorized users.

## Introduction

Type 2 diabetes mellitus is a large and growing healthcare problem that leads to macro- and microvascular complications [1]. Type 2 diabetes is a health threat to both men and women. Nonetheless, sex differences have been described in the occurrence of macrovascular complications, where women with type 2 diabetes are more often and worse affected than men [2]. So far, these differences have not resulted in sex-specific recommendations in the guidelines [3, 4]. It is uncertain whether sex differences exist in the prevalence and incidence of microvascular diseases in type 2 diabetes. Insights into differences between men and women may fuel development of more personalized treatment approaches. Existing studies show ambiguous outcomes, and not all microvascular outcomes have been investigated to the same extent. In a study by Stratton et al. [5], 6 years after diagnosis of type 2 diabetes, men had more diabetic retinopathy than women. In contrast, in a cross-sectional study of Korean patients, no sex difference was observed in prevalence rate of retinopathy [6]. Similar level of disparity has been reported on sex differences for neuropathy [7, 8] and albuminuria, reflecting microvascular renal disease [9–11]. Taken together, there is a lack of large prospective follow-up studies systematically investigating sex differences for each microvascular complication and the total burden of microvascular complications. We determined the sex difference in the incidence of each of the microvascular complications in the DiaGene study. In addition, we analyzed sex differences in simultaneous occurrence of multiple complications.

## Methods

### Study design

The design of the DiaGene study has been described previously [12]. In short, the DiaGene study is an all lines of care case–control study in Eindhoven and Veldhoven that was coordinated by the vascular section of the internal medicine department in the Erasmus MC. Virtually all type 2 diabetes patients in Eindhoven and Veldhoven were approached. Eventually 1886 patients with type 2 diabetes were included. Written informed consent was obtained from all participants. This study was approved by the Medical Ethics Committees of the Erasmus MC, Catharina Hospital and Maxima Medical Center.

### Definitions microvascular complications

Type 2 diabetes was defined in accordance with American Diabetes Association and World Health Organization guidelines, as described previously [13, 14]. Diabetic retinopathy was scored according to the report of an ophthalmologist as absent or present. If present, it was classified as non-proliferative, proliferative or retinopathy treated with photocoagulation or intra-vitreal injections. Neuropathy was diagnosed by a podotherapist, neurologist or the treating physician. Microalbuminuria as a reflection of microvascular renal disease was defined as (albumin creatinine ratio (ACR) ≥ 2.5 for men or ≥ 3.5 for women) at two of three consecutive measurements, or when high microalbuminuria or macroalbuminuria was present at one measurement (ACR ≥ 12.5 for men and ≥ 17.5 for women) [12].

### Clinical data

Laboratory data and anthropometrics were derived from medical records at inclusion. By means of a questionnaire, medical history, family history and lifestyle information was collected. Systolic blood pressure (SBP) > 140 mmHG and/or diastolic blood pressure (DBP) > 90 mmHg or use of antihypertensive therapy defined hypertension. Mean arterial pressure (MAP) was calculated with the formula (2 × diastolic pressure + systolic pressure)/3. Dyslipidemia was defined as LDL > 3.0 or usage of lipid-lowering medication. Non-HDL cholesterol was calculated with the formula non-HDL = total cholesterol-HDL cholesterol. Smoking status was defined as current smoker, former smoker or never smoker.

### Endpoints

Primary endpoints were each of the above-described microvascular complications: retinopathy, microalbuminuria and neuropathy. These microvascular complications were defined as absent or present. Neuropathy data were only available for outpatient hospital clinic patients. Analyses on neuropathy and the total burden of microvascular complications were therefore restricted to the hospital population (*n* = 830).

### Statistical analysis

To compare baseline variables, the independent samples T test was applied for continuous variables with a normal distribution and the Chi-square test for categorical variables. In cross-sectional analyses, applying multiple logistic regression models, odds ratio (ORs) and 95% confidence intervals (CIs) were estimated for the association between sex and prevalent microvascular complications. In the prospective analyses, using Cox proportional hazard models, hazard ratios (HRs) and 95% CIs were estimated to study the association between sex and first incident microvascular complication. All analyses were adjusted for potential confounders: age, smoking, MAP, dyslipidemia (non-HDL cholesterol and HDL cholesterol), HbA1c and duration of type 2 diabetes. In the prospective analyses, prevalent cases of the studied complication at baseline were excluded. To explore possible premenopausal hormonal effects, all analyses were repeated excluding women under the age of 51 years [15]. To investigate a possible interaction between sex and smoking, this interaction term was added as covariate in the models. To study the relationship of sex with the occurrence of multiple simultaneous complications at baseline and follow-up, ordinal logistic regression was performed. The dependent variable, number of microvascular complications was ordered in three categories, zero and one microvascular complications were classified as the reference category and two or three microvascular complications were the other separate categories. Additional models were corrected for risk factors for microvascular complications: age, smoking, MAP, dyslipidemia (non-HDL cholesterol and HDL cholesterol), HbA1c and duration of type 2 diabetes. Venn diagrams were created using R version 3.6.1 with package “Eulerr.” *p* < 0.05 was considered statistically significant. Statistical analyses were performed using SPSS version 22.0.

## Results

### Baseline patient characteristics

Baseline characteristics for men and women are shown in Table 1. Two patients were excluded because of unknown sex, leaving a total of 1884 patients for analyses. Women had a significantly longer time of follow-up (7.05 years vs. 6.83 years, *p* = 0.03), higher BMI (31.43 kg/cm^2^ vs. 29.63 kg/cm^2^, *p* < 0.001), higher HDL cholesterol (1.26 mmol/L vs. 1.09 mmol/L, *p* < 0.001), higher non-HDL cholesterol (3.21 mmol/L vs. 3.04 mmol/L, *p* < 0.001) and were older (65.82 years vs. 64.74 years, *p* = 0.026) compared to men. In contrast, men were significant more likely to be current (19% vs. 13%, *p* < 0.001) and former smokers (60% vs. 13%, *p* < 0.001) than women.

**Table 1:** Baseline characteristics of men and women in the DiaGene study

DiaGene	Men (*n* = 1006)	Women (*n* = 878)	*p* Value
Age (years)	64.74 (± 10.44)	65.82 (± 10.69)	0.026
Duration of diabetes (years)	9.84 (± 8.14)	10.38 (± 8.78)	0.179
Mean follow-up (years)	6.83 (± 2.19)	7.05 (± 2.05)	0.030
BMI (kg/m^2^)	29.63 (± 4.63)	31.43 (± 6.09)	< 0.001
MAP (mmHg)	99.25 (± 10.87)	98.47(± 10.75)	0.133
HbA1c (%)	7.02 (± 1.08)	7.07 (1.05)	0.326
HbA1c (mmol/mol)	53 (± 12)	53 (± 11)	0.326
Total cholesterol (mmol/L)	4.14 (± 0.88)	4.47 (± 0.95)	< 0.001
LDL cholesterol (mmol/L)	2.35 (± 0.79)	2.55 (± 0.85)	< 0.001
HDL cholesterol (mmol/L)	1.09 (± 0.30)	1.26 (± 0.32)	< 0.001
Non-HDL cholesterol (mmol/L)	3.04 (± 0.860)	3.21 (± 0.94)	< 0.001
Primary care (%)	562 (56)	494 (56)	0.862
Secondary care (%)	444(44)	384(44)	
Dyslipidemia (%)	563 (56)	530 (60)	0.100
Missing data (%)	91 (9)	67 (8)	
Smoking (%)			< 0.001
Never smoked	131 (13)	309 (35)	
Current smoker	190 (19)	115 (13)	
Former smoker	607 (60)	305 (40)	
Missing data (%)	78 (8)	104 (12)	
Diabetes medication (%)			
No	177 (18)	163 (19)	0.508
Insulin and analogues	297 (29)	275 (31)	0.309
Oral antidiabetics	616 (61)	524 (60)	0.653
Missing data (%)	55 (5)	57 (6)	
Use of antihypertensive medication (%)	662 (66)	591 (67)	0.295
Missing data (%)	55 (5)	57 (6)	
Use of lipid-lowering medication agents (%)	670 (66)	563 (64)	0.392
Missing data (%)	55 (5)	57 (6)	
Macrovascular complications (%)	415 (41)	245 (28)	< 0.001
Ischemic heart disease (%)	329 (33)	168 (20)	< 0.001
Ischemic brain disease (%)	128 (13)	83 (10)	0.019
Missing data (%)	57 (6)	76 (9)	
Microvascular complications (%)	402 (40)	262 (31)	< 0.001
Retinopathy (%)	184 (18)	124 (15)	0.032
Microalbuminuria (%)	263 (26)	124 (15)	< 0.001
Neuropathy (%)	126 (12)	112 (13)	0.727
Missing data (%)	138 (13)	146 (17)	

Unless stated otherwise, mean (± SD) are given

### Sex and prevalent complications at baseline

In cross-sectional analyses at baseline, men had more macro- and microvascular complications. Men had significantly higher age adjusted odds ratio for retinopathy [OR 1.36 (CI 1.06–1.75)] and microalbuminuria [OR 2.38 (CI 1.87–3.04)]. These associations remain significant for both retinopathy [OR 1.98 (CI 1.39–2.81)] and microalbuminuria [OR for men 1.85 (CI 1.37–2.49)] after adjustment for multiple confounders (Supplementary Table 1). No significant differences between the sexes were found for neuropathy at baseline. There was no significant interaction between smoking and sex for all microvascular complications. Also excluding women under the age of 51 years from the analyses did not substantially change these results (Supplementary Table 2).

### Sex and incident complications at follow-up

Table 2 shows the Cox proportion hazard models for prospective analyses. No significant differences were found in retinopathy and neuropathy. Men had a significant higher hazard ratio for microalbuminuria [HR 1.64 (CI 1.21–2.24)] in both models. There was no significant interaction between smoking and sex on the outcomes of retinopathy and neuropathy. For microalbuminuria, a significant association of this interaction term was seen in both models [HR 0.60 (CI 0.38–0.96)], indicating a more detrimental effect of smoking in women. Excluding women under the age of 51 years from the analyses did not substantially change these results (Supplementary Table 3).

**Table 2:** Hazard ratios for incident microvascular complications according to sex

	Model 0 HR	95% CI	*p* Value	Model 1 HR	95% CI	*p* Value
Retinopathy Men	1.13	0.87–1.47	0.360	1.27	0.93–1.74	0.130
Microalbuminuria Men	1.89	1.46–2.44	< 0.001	1.64	1.21–2.24	0.002
Neuropathy Men	1.28	0.99–1.65	0.061	1.35	0.99–1.83	0.057

Women are the reference group

Model 0: adjusted for age

Model 1: additionally adjusted for smoking, HbA1c, MAP, non-HDL cholesterol, HDL cholesterol and duration of diabetes * Adding covariates interaction term sex * smoking, line of care and BMI did not change results significantly

*HR* hazard ratio, *CI* confidence interval

### Total burden of complications in men and women at baseline and follow-up

**Fig. 1:**
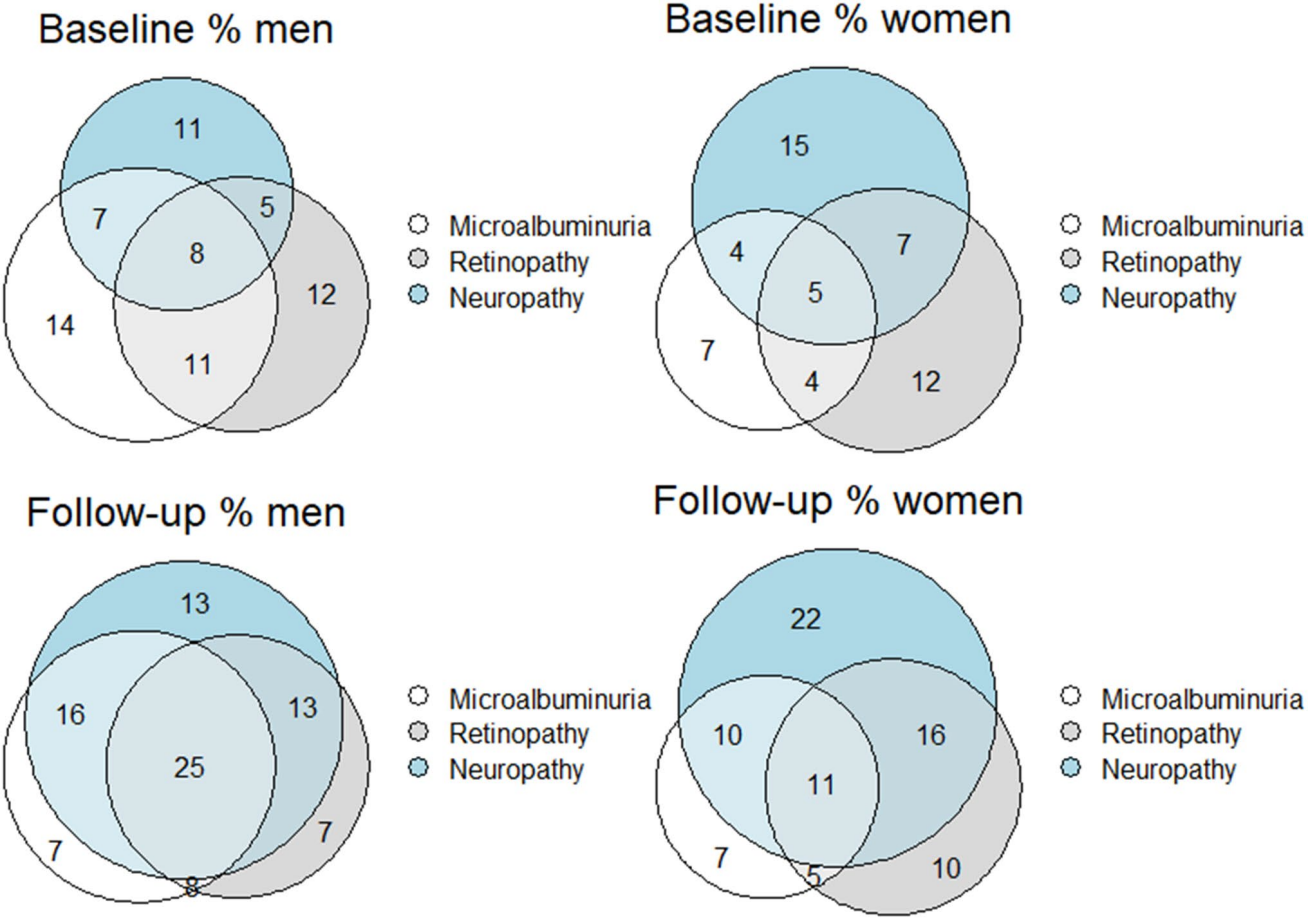
Overlap microvascular complications at baseline and follow-up in outpatient clinic patients according to sex

At baseline and follow-up, men were significantly more likely than women to have two or three microvascular complications also after correction for conventional risk factors (Table 3) (OR 2.42 [CI 1.69–3.45] at follow-up for the fully adjusted model). Figure 1 illustrates the overlap of complications in men and women at baseline and follow-up, only for the secondary care patients.

**Table 3:** Ordinal regression of microvascular complications at baseline and follow-up

	Baseline OR	95% CI	*p* Value	Follow-up OR	95% CI	*p* Value
Model 0 Men	1.73	1.23–2.43	0.002	2.45	1.83–3.29	< 0.001
Model 1 Men	1.38	1.01–2.32	0.046	2.42	1.69–3.45	< 0.001

Women are the reference group

Model 0: adjusted for age

Model 1: additionally adjusted for smoking, HbA1c, MAP, non-HDL cholesterol, HDL cholesterol and duration of diabetes

*OR* odds ratio, *CI* confidence interval

## Discussion

In our large prospective cohort study of patients with type 2 diabetes, we found sex differences in microvascular complications at baseline and at prospective follow-up, with men being more affected than women. At baseline, men had a significantly higher frequency of microalbuminuria and retinopathy. Prospective analyses showed that men are more at risk of developing microalbuminuria. In addition, men were more likely to develop multiple microvascular complications, while women more often had one isolated complication.

In contrast to macrovascular disease, little is known about sex differences in microvascular complications in type 2 diabetes due to paucity of prospective data. Therefore, we decided to perform this prospective study, with ample information on all microvascular complications and confounding factors. The results of our study are partly in line with previous retrospective, cross-sectional and prospective studies on sex difference in microvascular complications. Regarding retinopathy, a cross-sectional study by Looker et al. [16] found that retinopathy was associated with male sex after correcting for risk factors. Also prospective analyses by Semeraro et al. [17] and Stratton et al. [5] showed significant positive associations of retinopathy with male sex. Interestingly, Ozawa et al. [18] found the relationship between diabetic retinopathy and men appears to weaken with longer duration of type 2 diabetes. We did not find significant differences in the incidence of retinopathy between men and women, although retinopathy was more prevalent in men at baseline. For albuminuria, previous cross-sectional studies reported a higher prevalence in men [19, 20]_._ Prospective studies have shown conflicting results reporting positive [9], negative [21] and absent [22] associations of male sex with albuminuria. Regarding neuropathy, in Caucasians a higher prevalence in men was found [7]. In contrast, in Asians, a higher prevalence of neuropathy among women was reported [8]. Of note, these studies did not correct for height, which was shown to be more strongly associated with neuropathy than sex [23]. We could unfortunately also not correct for height, as we did not have this available as an independent measurement. Part of the higher prevalence in retinopathy and incidence and prevalence in microalbuminuria in men may be explained by differences in risk factors for these complications between men and women. In our study, men were more often current and former smokers. In contrast, women had higher total, LDL and non-HDL cholesterol levels. These findings are in line with other studies [24, 25]. However, after correction for these risk factors, the sex effect on microvascular disease remained. Additional analyses, and also adjusting for other risk factors such as BMI and line of care in the models did not change the results substantially (data not shown). Because men were more often current and former smoker, we investigated a potential interaction of smoking and sex. This interaction was nonsignificant for the majority of outcomes. In microalbuminuria, it showed that smoking in women is associated with a higher risk. This effect therefore does not explain the higher risk of nephropathy in men. It therefore seems unlikely that smoking explains these differences, but we cannot exclude a residual undetectable level of microvascular damage due to differences in smoking habits and smoking history between men and women at baseline. As men and women were represented in equal proportions in primary and secondary care and the guidelines for treatment in these lines of care were identical, this also does not seem to play a role in the findings, unlike what was observed elsewhere [26]. Taken together, these findings suggest that residual uncorrected differences or yet unknown factors may play a role. One of these may be the difference in sex hormones. For macrovascular complications, it is known that men and postmenopausal women have a higher risk of cardiovascular disease compared to premenopausal women, which suggests a protective effect of endogenous female hormones such as estrogen and progesterone [27]. Several studies have reported that lower testosterone levels in men with type 2 diabetes are associated with macrovascular complications and that higher testosterone levels in men are associated with lower cardiovascular disease (CVD) and all-cause mortality [28, 29]_._ However, the association of sex hormones with microvascular complications is unknown. To investigate a possible hormonal explanation, we repeated our analyses excluding women younger than 51 years of age. We observed no substantial changes in the results. These analyses take sex hormones differences as the definite explanation unlikely. However, it cannot be ruled out that the difference in premenopausal exposure to sex hormones between men and women may partly account for our findings.

Not only sex differences but also gender differences in behavior and treatment between men and women may be involved in the differences in outcomes. A study of Kramer et al. [30] observed that men are treated more intensively for type 2 diabetes and CVD, which may result in earlier diagnosis and treatment of complications. Another study observed less effective treatment of lipid disorders in women [31]_._ These differences would, however, be expected to result in a lower rather than higher microvascular complication rate in men. Moreover, the participants in our study were all treated according to the current guidelines for type 2 diabetes and prevention of complications. We have no indications from our data that women were treated less stringent. They were treated with lipid-lowering therapy, antidiabetic medication and antihypertensive medication in equal rates. We cannot exclude differences in medications adherence between men and women. There are several studies that conclude that women have a lower medication adherence compared to men [32]_._ This may be a possible explanation of the significant differences in lipid status of men and women in our study, but does not explain a higher microvascular complication rate in men. Strengths of our study are the prospective study design, large study size and meticulous collection of phenotypic, medication and risk factor data. Although we have performed our study with great care, we need to consider some limitations. First, stages of diabetic nephropathy are defined based on magnitude of albuminuria and kidney function estimated by eGFR. Diabetic nephropathy can follow a non-albuminuric pathway to renal function loss. However, non-albuminuric diabetic nephropathy is associated with a higher prevalence of CVD and may be more reflective of macroangiopathy than microangiopathy, which is the subject of this study [33]. In the present study, we analyzed renal disease based on albuminuria. This means that individuals with non-albuminuric diabetic nephropathy are not taken into account in our analyses. Second, neuropathy data were only available for patients that were under surveillance in the hospitals. This reduced power for these analyses and generalizability to non-hospital patients. Also, height is a risk factor for neuropathy that we did not have available for correction in our models. Third, participation bias needs to be considered; however, we collected from both primary and secondary care, and in both of these populations, high rate of complications was found. Therefore, a “healthy volunteer” bias seems unlikely. Finally, to investigate whether sex hormones played a role we repeated our analyses excluding women below the age of 51. This is a surrogate way of excluding premenopausal women and does not exclude residual effects of sex hormones on the outcome. Unfortunately we have no measures of sex hormones available in our study population to investigate this in more detail. In conclusion, we found that men are at a higher prospective risk of several microvascular complications even after correcting for conventional risk factors. This underlines that men and women may not benefit to a similar extent from current treatment approaches to prevent microvascular complications. Future studies should be directed at investigating the underlying mechanism of this association and to use this knowledge for improving personalized treatment strategies to prevent microvascular complication in type 2 diabetes.

## Electronic supplementary material

Below is the link to the electronic supplementary material.

Supplementary file1 (DOCX 19 kb)

## Data Availability

The datasets generated during and/or analyzed during the current study are not publicly available. The raw data are subject to “Special Categories of Personal Data (Sensitive Data)” (GDPR, Article 9); therefore, raw data sharing is not in line with the privacy principles. Also, the information provided to the participants in the study states that the individual data are only accessible to the researchers, the ethical review board and (local) authorities. The informed consent given by the participants is therefore not sufficient for open access publication of indirectly identifiable data. Datasets are available from the corresponding author upon reasonable request.
