# Supplementary material for: Sex difference in the incidence of microvascular complications in patients with type 2 diabetes mellitus: a prospective cohort study
Source: Acta Diabetol. 2020 Feb 5;57(6):725–32. doi: 10.1007/s00592-020-01489-6 (PMC7220974; doi:10.1007/s00592-020-01489-6)
Supplement: Supplementary file 1 — Supplementary file1 (DOCX 19 kb) [file 592_2020_1489_MOESM1_ESM.docx]

**Title**: Sex difference in the incidence of microvascular complications in patients with type 2 diabetes mellitus

A prospective cohort study

**Journal:** Acta Diabetologica

**Authors:** Sunny S. Singh , Jeanine Roeters-van Lennep, Roosmarijn Lemmers , Thijs van Herpt, Aloysius G. Lieverse, Eric J.G. Sijbrands, Mandy van Hoek

**Corresponding author:**

Mandy van Hoek

M.vanhoek@erasmusmc.nl

ORCID-ID: 0000-0002-2957-5436

**Supplementary tables**

**Supplementary table 1**: Odds ratio for prevalent microvascular complications according to sex

|  | **Model 0**  **OR** | **95 % CI** | **P value** | **Model 1**  **OR** | **95 % CI** | **P value** |
| --- | --- | --- | --- | --- | --- | --- |
| ***Retinopathy***  **Men** | **1.36** | 1.06-1.75 | 0.02 | **1.98** | 1.39-2.81 | <0.001 |
| ***Microalbuminuria***  **Men** | **2.38** | 1.87-3.04 | <0.001 | **1.85** | 1.37-2.49 | <0.001 |
| ***Neuropathy***  **Men** | **1.01** | 0.74-1.39 | 0.939 | **0.96** | 0.65-1.42 | 0.845 |

**_Abbreviation:_** _OR, odds ratio; CI, confidence interval_

***_Women are the reference group._***

_Model 0: Adjusted for age_

_Model 1: Additionally adjusted for smoking, HbA1c, MAP, non-HDL-cholesterol, HDL-cholesterol and duration of diabetes * Adding covariates interaction term sex*smoking, line of care and BMI didn’t change results significantly_

**Supplementary table 2:** Odds ratio for prevalent microvascular complications according to sex, excluding women aged 51 years or younger

|  | **Model 0**  **OR** | **95 % CI** | **P value** | **Model 1**  **OR** | **95 % CI** | **P value** |
| --- | --- | --- | --- | --- | --- | --- |
| ***Retinopathy***  **Men** | **1.38** | 1.07-1.79 | 0.014 | **2.04** | 1.42-2.94 | <0.001 |
| ***Microalbuminuria***  **Men** | **2.38** | 1.85-3.05 | <0.001 | **1.89** | 1.40-2.57 | <0.001 |
| ***Neuropathy***  **Men** | **0.98** | 0.71-1.36 | 0.907 | **0.97** | 0.65-1.45 | 0.881 |

**_Abbreviation:_** _OR, odds ratio; CI, confidence interval_

***_Women are the reference group._***

_Model 0: Adjusted for age_

_Model 1: Additionally adjusted for smoking, HbA1c, MAP, non-HDL-cholesterol, HDL-cholesterol and duration of diabetes * Adding covariates interaction term sex*smoking, line of care and BMI didn’t change results significantly_

**Supplementary table 3:** Cox Hazard regression analyses corrected for co-variables, excluding women aged 51 years or younger

|  | **Model 0**  **HR** | **95 % CI** | **P value** | **Model 1**  **HR** | 95 % CI | **P value** |
| --- | --- | --- | --- | --- | --- | --- |
| ***Retinopathy***  **Men** | **1.10** | 0.84- 1.44 | 0.488 | **1.22** | 0.88-1.69 | 0.228 |
| ***Microalbuminuria***  **Men** | **1.90** | 1.47- 2.46 | <0.001 | **1.59** | 1.16-2.17 | 0.004 |
| ***Neuropathy***  **Men** | **1.25** | 0.97- 1.63 | 0.09 | **1.34** | 0.98-1.83 | 0.067 |

**_Abbreviation:_** _OR, odds ratio; CI, confidence interval_

***_Women are the reference group._***

_Model 0: Adjusted for age Model 1: Additionally adjusted for smoking, HbA1c, MAP, non-HDL-cholesterol, HDL-cholesterol and duration of diabetes * Adding covariates interaction term sex*smoking, line of care and BMI didn’t change results significantly_
